# Nurse competence in the interface between primary and tertiary healthcare services

**DOI:** 10.1002/nop2.230

**Published:** 2018-12-13

**Authors:** Ann‐Chatrin Linqvist Leonardsen, Annette Bjerkenes, Inga Rutherford

**Affiliations:** ^1^ Østfold University College/Østfold Hospital Trust Grålum Norway; ^2^ Helsehuset‐Indre Østfold Medical Competence Centre Askim Norway; ^3^ Peer Gynt Helsehus‐ Municipal Acute Ward Moss Norway

**Keywords:** healthcare quality, job satisfaction, nurse competence, patient safety, primary and tertiary health care, self‐assessed competence

## Abstract

**Aims:**

(a) To explore nurses’ self‐assessed competence and perceived need for more training in primary and tertiary healthcare services; and (b) to investigate the factors associated with these issues.

**Design:**

Quantitative, cross‐sectional, descriptive.

**Methods:**

The ProffNurseSAS, the Job Satisfaction Scale and socio‐demographics were used. A convenient sampling method was used to invite registered nurses from 23 primary (*N* = 104) and tertiary care wards (*N* = 26).

**Results:**

Five significant differences in self‐assessed competence were identified, with none regarding the perceived need for more training between nurses working in primary versus tertiary health care. Nurses in primary health care had longer experience, and a larger proportion had continuing education. Nevertheless, this was not associated with either self‐assessed competence or the perceived need for more training. Years of experience, training or reported job satisfaction was not associated with the items on the ProffNurseSAS.

**Conclusion:**

Findings indicate that nurses’ competence is same in primary and tertiary healthcare settings. Moreover, the findings of this research highlight areas that need further improvement and emphasis from both leaders and educational institutions when they attempt to ensure nurses’ competence.

## 1 INTRODUCTION

Healthcare reforms and innovative policies have been introduced worldwide to meet the upcoming challenges due to an increased number of elderly people and people with chronic diseases (St. Sauer, 2015; Uijen & van de Lisdonk 2008). The healthcare services in Norway are divided into two levels. The state is responsible for tertiary healthcare services provided in specialist hospitals, outpatient services and emergency services, while the municipalities are responsible for primary health care, including general practice, home‐based care and nursing homes. In Norway, the implementation of the Coordination Reform of 2012 has led to a greater number of patients receiving more specialized medical treatments outside hospitals (The Norwegian Department of Health & Care, 2009). For example, the establishment of municipal acute wards (MAWs) has led to patients who earlier were hospitalized now receiving acute healthcare services in the municipalities. Moreover, there has been an increased development and/or new establishment, of intermediate care units for patients before and/or after a hospital stay. These wards are called *units for patients ready for discharge* or *short‐stay wards* (Johannessen, Luras, & Steihaug, 2013; Lappegard & Hjortdahl, 2012; Romoren, Torjesen, & Landmark, 2011). Similar intermediate care wards have been implemented internationally to meet future healthcare challenges. They aim to ensure the integration of services and collaboration in and between primary and tertiary healthcare settings (Grimsmo & Magnussen, 2015; Smith et al., 2013). Intermediate care units have existed both nationally and internationally for several years in, for example, so‐called general practitioner hospitals (GPHS) or cottage hospitals (Aaraas, 1998). Nevertheless, the increasing focus on the decentralization, coordination and integration of services has led to more and more patients also receiving active medical treatment outside hospitals (Lillebo, Dyrstad, & Grimsmo, 2013; Swanson & Hagen, 2016). Healthcare services are exposed to efficiency demands, a focus on early discharge from pressured hospitals and a lack of financial resources (OECD, 2010, 2013). These demands often lead to political initiatives that are not necessarily built on professionals’ and/or patients’ evaluations of what characterizes safe, quality healthcare services.

The extensive development and complexity of healthcare services challenge the competence of healthcare workers (World Health Organization, 2010). International organizations have emphasized the importance of nurses’ education and competence to ensure quality and patient safety in healthcare services (Institute of Medicine, 2003; International Council of Nurses, 2012). Competence has been described as a combination of knowledge, fitness, assessments and attitudes, but there is no consensus on a definition of “nursing competence” (Cowan, Norman, & Coopamah, 2005; Cowan, Wilson‐Barnett, Norman, & Murrells, 2008). The World Health Organization describes “nurses’ professional competence” as a framework of skills that reflects knowledge, attitudes, as well as psychosocial and psycho‐motor elements (World Health Organization, 2009).

Nurses report that they need to increase their knowledge in, for example, pharmacology and age‐related physiological changes. Nevertheless, they report a lack of training and education in such areas (Simonsen, Daehlin, Johansson, & Farup, 2014; Simonsen, Johansson, Daehlin, Osvik, & Farup, 2011). The decentralization of specialist/tertiary healthcare services and the complexity of patient cases challenge the knowledge, training and competencies of nurses working in primary healthcare services. For example, there has been an increased use of medical–technical devices outside hospitals (Gautun & Syse, 2013). Studies from GPHS (Aaraas, 1998), intermediate care units (Garåsen, 2008) and community hospitals (Lappegard, 2016) indicate that the decentralization of specialist healthcare services does not necessarily have an impact on patient safety and quality, as measured by patient outcomes.

The importance of nurses’ competence in ensuring patient safety has been confirmed in several studies (Finnbakk, Wangensteen, Skovdahl, & Fagerström, 2015; Kirwan, Matthews, & Scott, 2013; Needleman & Hassmiller, 2009), as well as its connections to healthcare quality (Naylor et al., 2013). Nevertheless, we could not identify studies that explore nurses’ competence in the newly established primary healthcare wards.

## 2 AIM

This study purported to explore and compare nurses’ self‐assessed competence, as well as their perceived need for more training and education in primary and tertiary healthcare services, respectively. Moreover, the aim of this work was to explore factors associated with these issues, such as age, gender, continuing education, years of experience as a nurse, years of experience in primary health care, years of experience in hospitals, targeted training during the last two years and job satisfaction. An assessment of nurse competence might be used to promote professional development, to adjust nurses’ competencies to public needs and to assess organizational performance (Hamström, Kankkunen, Suominen, & Meretoja, 2012).

## 3 METHODS

### 3.1 Design

This study had a cross‐sectional, descriptive, quantitative design, using a questionnaire to explore nurses’ competence in primary and tertiary health care.

### 3.2 Setting and participants

This study was conducted in a county in the southeastern part of Norway, with approximately 290,000 inhabitants. Registered nurses were invited from all the primary healthcare wards that treated patients who earlier were hospitalized: MAWs, units for patients ready for discharge, short‐stay wards and rehabilitation and palliation wards (*N* = 20). Table 1 gives an overview of the included primary healthcare wards.

**Table 1:** Primary healthcare wards included

	Sarpsborg (*N* = 5)	Halden (*N* = 2)	Indre Østfold (*N* = 5)	Fredrikstad (*N* = 4)	Moss (*N* = 4)	Total *N* = 20
Municipal acute ward	8	5	7	11	10	41
Rehabilitation ward	6	20	21	12	8	67
Palliation ward	4	6	8	–	5	23
Short‐stay ward	54	17	28	96	20	215
Mixed ward	13					13
Total	85	48	64	119	43	359

Note

*N*: number of included wards; Numbers in table: number of beds in the ward(s).

The wards are distributed in accordance with the establishment of the five MAWs in the county, in the five different municipalities containing the head city. In addition, nurses from three wards in the county hospital were invited to participate. These wards were selected due to treating patients considered similar to and representative of patients in primary health care, namely an infection ward (beds = 24), a geriatric ward (beds = 18) and an observation ward (beds = 22).

A convenient sampling method was used: all the nurses fulfilling the inclusion criteria in the selected wards were invited to participate. Consequently, we did not conduct sample size calculations. Inclusion criteria were as follows: registered nurses with a minimum of one‐year experience at their present job, nurses with a minimum of 50% clinical work with direct patient contact and nurses with sufficient Norwegian fluency to understand and respond to the questionnaire.

### 3.3 Data collection

The questionnaire consisted of three different parts:

Part 1: Demographics, which included information about gender, age, percentage of employment, educational background, experience as a nurse, experience with primary health care, experience with tertiary health care, type of ward and targeted training conducted during the past two years.

Part 2: The professional nurse self‐assessment scale, ProffNurseSAS (Finnbakk et al., 2015), consisting of 50 questions. The development of the questionnaire was influenced by the validated questionnaire the Nurse Competence Scale (NCS) (Meretoja, Isoaho, & Leino‐Kilpi, 2004). The ProffNurseSAS includes information regarding nurses’ clinical practice, professional development, ethical decision‐making, clinical leadership, cooperation and consultation and critical thinking. Nurses are asked to: (a) assess their own competence; (b) to evaluate their need for more training and education; and (c) to report whether this item was covered in their nursing educational programme (yes/no) related to each of the 50 questions in the questionnaire (Finnbakk et al., 2015). The questionnaire uses a 10‐point numeric rating scale on the (a) and (b) items, respectively (1 = lack of competence/no need for further training or education, 10 = excellent competence/extensive need for further training or education).

Part 3: The Job Satisfaction Scale (JSS) is used to map job satisfaction (Andersen & Andersen, 2012; Warr, Cook, & Wall, 1979). The JSS builds on 10 aspects of working conditions: responsibility, variation in tasks, relationship to colleagues, physical environment, opportunity to use one's own abilities, summated job satisfaction, freedom to decide one's working methods, acknowledgement, income or wages and working hours. The scale is scored on a 7‐point Likert scale, where 1 = very dissatisfied and 7 = very satisfied.

Informational meetings with nurses and leaders were conducted in all the wards (*N* = 23) before data collection, in addition to information about the study sent by email. The questionnaires were distributed on paper and collected in sealed boxes at each ward, during three weeks in March 2018.

### 3.4 Statistical analysis

Frequencies were used to present characteristics of the study sample. Continuous variables were summarized by their median, mean and standard deviation (*SD*). Both mean and median were reported to show the skewness in the data. Since data were skewed (not normally distributed), the Mann–Whitney U test was used to compare primary and tertiary care wards. A multiple generalized linear regression model was used to identify the association between self‐assessed competence; the perceived need for more training (=dependent variables); and the covariates age, gender, ward, education level, years of experience as a nurse, years of experience in primary health care, years of experience in a hospital, training conducted for the past two years and score on the Job Satisfaction Scale (=independent variables). A significance level of 0.05 was chosen. All analyses were performed using the Statistical Package for the Social Sciences, SPSS Version 21 (IBM Corporation, 2012). The internal consistency of the items was analysed by calculating the Cronbach's alpha coefficient. No methods for calculating missing items exist.

### 3.5 Ethical considerations

Approval was sought and given by the Regional Committee for Medical and Healthcare Research (REK; Ref. no. 2017/2177‐3), as well as the Norwegian Center for Research Data (Ref. no. 56640). Approval and consent to participate were collected from the leaders of all the participating wards. Participation was based on guidelines for ethical research in the Declaration of Helsinki (World Medical Association, 2015) and on willing, informed consent. A returned, completed questionnaire was considered a written consent to participate. Since data were unidentifiable, nurses had no opportunity to withdraw from the study after returning their questionnaire. Data were handled anonymously and confidentially, and participants are not recognizable in the presentation of the study's findings. Data were kept in the research area of a safe, internal zone (mandating password and user access) at the hospital trust.

## 4 RESULTS

A total of 245 nurses in primary healthcare wards fulfilled the inclusion criteria. Of these, 104 (42.4%) completed the questionnaire. In the hospital wards, 75 nurses fulfilled the criteria and 26 (34.7%) responded. The percentage of responses differed in the primary healthcare wards between 22–100 and in the hospital wards between 25–65.

### 4.1 Sample

Nurses’ demographics (percentage of employment, years since graduation from nursing school, years of experience in primary and tertiary health care, respectively, continuing education after bachelor's degree in nursing, training for the past two years and training or education related to a specific patient case) are presented in Table 2.

**Table 2:** Description of participant demographics

	Primary health care (*N* = 104)	Tertiary helath care (*N* = 26)	*p*‐Value
Percentage of employment	87.6% (97.3) *SD* = 14.9	100% (100)	0.48
Years since graduated nurse	2.9 (4.0) *SD* = 1.2	1.9 (2.0)	<0.001
Years of experience from primary health care	2.4 (2.0) *SD* = 1.2	1.5 (1.0) *SD* = 0.8	<0.01
Years of experience from tertiary health care	1.8 (1.0) *SD* = 1.1	1.5 (1.0) *SD* = 0.8	0.26
Continuing education	50%	15.4%	<0.001
Further training last two years	88.5%	96.2%	0.24
Training in a specific patient case	59.6%	46.2%	0.22

Note

*SD*, standard deviation. Median in parentheses. Based on Mann–Whitney U test.

Nurses in primary health care had significantly longer experience as a nurse and more years of experience from primary health care. A larger proportion of the nurses had continuing education after their bachelor's degree than tertiary care nurses (Table 2). Nurses’ continuing education is presented in Table 3.

**Table 3:** Overview of nurses’ continuing education and courses in primary and tertiary health care

	Continuing education				Courses	
	Management	A/I/AC	Master's degree	Pedagogics	ABCDE	HHLR
Primary health care	20.9%	18.8%	8.3%	6.3%	59.8%	32.5%
Tertiary health care		50.0%			91.7%	8.3%

Note

A/I/AC: continuing education in anaesthesia, intensive care, acute care; ABCDE: courses in Airways–Breathing–Circulation–Examination, such as ProAct or Alert; HHLR: course in heart and lung resuscitation for healthcare personnel.

Training for specific patient cases was mainly conducted in primary healthcare nurses. This education included ventilator treatment (*N* = 16), care for patients with tracheostomies (*N* = 16), end‐of‐life treatment (*N* = 6) and peritoneal dialysis (*N* = 4). In tertiary health care, such specific training was related to the use of various medical–technical equipment.

### 4.2 ProffNurseSAS

Comparative analyses, as assessed by the Mann–Whitney U test, showed only six significant differences in self‐assessed competence on the 50 items of the ProffNurseSAS between nurses in primary and tertiary healthcare services. An overview of responses to each of the 50 items is presented in Table 4.

**Table 4:** Nurse scores on the ProffNurseSAS

Question/item	Self‐assessed competence (mean/*SD*)		*p*‐value	Need more training (mean/*SD*)		*p*‐value	Not covered in educational programme
	Primary (*N* = 104)	Tertiary (*N* = 26)		Primary (*N* = 104)	Tertiary (*N* = 26)		
I am independently responsible for health assessment (systematic physical examination), examinations and treatment of patients with complicated medical conditions	7.38 (1.5)	7.4 (1.4)	0.95	5.9 (2.5)	5.6 (1.9)	0.67	*N* = 18
I am independently responsible for health assessment (systematic physical examination), examinations and treatment of patients with uncomplicated medical conditions	8.4 (1.3)	8.0 (1.2)	0.10	4.4 (2.7)	5.0 (2.6)	0.25	*N* = 9
I plan and prioritize nursing and medical interventions	8.3 (1.3)	8.6 (1.0)	0.56	4.7 (2.6)	4.8 (2.3)	0.75	*N* = 8
I identify patient's health problems	8.3 (1.2)	8.0 (1.3)	0.33	4.9 (2.8)	5.0 (2.2)	0.86	*N* = 4
I assess patient's symptoms	8.6 (1.2)	8.7 (1.1)	0.85	4.9 (2.9)	4.5 (2.4)	0.64	*N* = 4
I evaluate and modify patients’ medical treatment	7.5 (1.8)	7.2 (2.3)	0.68	5.3 (2.5)	5.2 (2.7)	0.98	*N* = 16
I exclude differential diagnoses when assessing patients’ health conditions	6.9 (1.8)	6.1 (2.5)	0.16	5.7 (2.6)	5.8 (2.5)	0.97	*N* = 16
I interpret, analyze and reach alternative conclusions about patients’ health conditions after a detailed mapping of health history and health assessment (physical examination)	7.1 (1.8)	7.3 (2.0)	0.49	5.7 (2.7)	5.3 (2.2)	0.46	*N* = 15
I apply both subjective and objective methods when examining, treating and caring for patients	7.9 (1.3)	7.6 (1.2)	0.29	5.3 (2.8)	5.5 (2.6)	0.79	*N* = 8
I utilize medical equipment in an appropriate and accurate manner	8.8 (1.2)	8.5 (1.1)	0.19	4.9 (2.9)	4.5 (2.3)	0.79	*N* = 12
I have knowledge of the effects of medication and treatment for the patients I am responsible for	7.8 (1.5)	7.6 (1.9)	0.91	5.8 (2.8)	6.1 (2.8)	0.40	*N* = 9
I identify deviations in the patients' state of health and state of disease	8.2 (1.3)	8.4 (1.0)	0.40	4.9 (2.8)	5.0 (2.7)	0.83	*N* = 2
I develop and administer health‐promoting and illness‐preventive actions for patients	7.8 (1.5)	7.9 (1.4)	0.33	4.8 (2.7)	5.6 (2.7)	0.36	*N* = 3
I systematically gather information from each patient about his/her health resources	6.6 (1.9)	8.2 (1.3)	0.38	6.7 (2.7)	4.4 (2.5)	0.56	*N* = 8
I have knowledge of the interactions of various types of medication and what side‐effects they may cause for the patients I am responsible for	6.9 (2.1)	6.1 (1.7)	0.22	5.3 (2.6)	7.4 (2.1)	0.35	*N* = 17
I generate a creative learning environment for staff at my workplace	6.6 (2.6)	6.7 (1.7)	0.43	5.4 (2.8)	5.5 (2.7)	0.71	*N* = 17
I participate in quality development work at my workplace	6.9 (2.5)	6.9 (2.2)	0.83	5.6 (2.7)	5.5 (2.3)	0.76	*N* = 16
I take responsibility for competence development at my workplace	6.9 (2.4)	6.3 (2.3)	0.33	5.3 (2.5)	5.9 (2.2)	0.58	*N* = 14
I improve routines/systems that fail to meet the needs of patients at my workplace	8.3 (1.5)	6.5 (2.0)	0.43	5.7 (2.8)	6.0 (2.6)	0.33	*N* = 19
I take active responsibility for my own professional development	8.4 (1.3)	7.9 (1.8)	0.39	5.2 (2.7)	6.3 (2.2)	0.30	*N* = 5
I take patients’ mental health needs (mood swings, feelings of hopelessness, depression, etc.) into account when assessing and planning for the health and life situation of patients	8.1 (1.7)	7.7 (1.6)	0.04*	5.3 (2.7)	5.9 (2.2)	0.15	*N* = 6
I take patients’ spiritual health needs (feelings of meaninglessness, existential needs, beliefs, fear of death, etc.) into account when assessing and planning for the health and life situation of patients	8.8 (1.0)	7.3 (1.9)	0.04*	4.9 (2.9)	5.7 (2.3)	0.46	*N* = 6
I take patients’ physical health needs (illness, pain, disabilities, etc.) into account when assessing and planning for the health and life situation of patients	9.0 (1.1)	8.3 (1.2)	0.04*	4.3 (2.7)*	5.5 (2.3)	0.37	*N* = 1
I adopt an ethical approach in my relationship with patients	8.5 (1.3)	8.8 (1.2)	0.27	4.4 (2.5)	5.1 (2.4)	0.16	‐
I identify and assume responsibility for patients’ own health resources in planning nursing care	7.1 (2.1)	8.3 (1.2)	0.42	4.6 (2.4)	5.0 (2.3)	0.24	*N* = 2
I take patients’ social health needs (leisure activities, friends, financial situation, etc.) into account when assessing and planning for the health and life situation of patients	8.4 (1.2)	5.9 (2.0)	<0.01*	4.7 (2.6)*	5.8 (2.2)	0.04*	*N* = 6
I support and guide patients in mastering their illnesses and health problems	8.6 (1.4)	8.0 (1.5)	0.45	4.0 (2.8)	5.0 (2.6)	0.72	*N* = 4
I maintain an ethical approach towards my colleagues	8.8 (1.1)	8.1 (1.8)	0.31	3.7 (2.7)	4.5 (2.4)	0.28	*N* = 4
I take active responsibility for creating a good working environment	8.6 (1.1)	8.8 (1.2)	0.79	4.4 (2.8)	4.1 (3.0)	0.68	*N* = 6
I put emphasis on patients’ own wishes when assessing and planning for nursing care and medical treatment	8.5 (1.3)	8.3 (1.4)	0.29	4.1 (2.7)	4.6 (2.5)	0.53	*N* = 3
I act ethically when caring for patients	9.5 (0.8)	7.9 (1.2)	0.01*	3.4 (2.9)	5.3 (2.8)	0.08	*N* = 7
I take full responsibility for my own actions	8.6 (1.2)	9.4 (0.8)	0.16	4.1 (2.9)	3.7 (2.8)	0.31	*N* = 6
I am correct and accurate in speech and writing	9.1 (1.0)	8.5 (1.2)	0.61	3.8 (2.8)	4.8 (2.9)	0.23	*N* = 6
I understand the consequences my decisions may have for patients	8.5 (1.8)	8.9 (1.1)	0.59	4.1 (3.1)	4.3 (2.7)	0.36	*N* = 4
I experience a division of responsibility between the physician and me as a nurse	8.9 (1.3)	9.0 (1.1)	0.43	3.1 (2.8)	4.3 (2.7)	0.26	*N* = 6
I cooperate well with the physician	8.8 (1.6)	8.2 (1.8)	0.38	3.4 (2.8)	4.8 (2.8)	0.51	*N* = 10
I consult other professional experts when required	9.0 (1.2)	8.8 (1.1)	0.80	3.4 (2.8)	4.1 (2.7)	0.10	*N* = 6
I cooperate actively with other health professionals when coordinating the patient's nursing, care and treatment	9.3 (0.9)	8.7 (1.5)	0.83	3.8 (3.3)	4.5 (3.0)	0.12	*N* = 5
I am cognizant of when my medical knowledge is insufficient when assessing patients’ health conditions	8.8 (1.1)	8.8 (1.8)	0.13	4.0 (2.9)	4.4 (3.2)	0.29	*N* = 4
I document the steps taken in assessing patients’ needs for nursing, care and treatment	9.1 (0.9)	8.9 (1.3)	0.19	3.8 (2.8)	4.2 (2.5)	0.78	*N* = 2
I reflect on my actions	9.1 (0.9)	8.4 (1.4)	0.70	3.9 (2.8)	4.1 (2.7)	0.24	*N* = 2
I analyze and evaluate my work continuously	8.7 (1.2)	9.0 (1.0)	0.28	4.1 (2.9)	4.5 (2.6)	0.33	*N* = 3
I perceive opportunities and have visions for how nursing and clinical paths for patients can be developed	8.1 (1.5)	8.4 (1.4)	0.75	4.5 (2.9)	4.6 (2.6)	0.53	*N* = 4
I have a vision of how nursing should be developed at my workplace	7.9 (1.9)	8.0 (1.6)	0.12	4.3 (2.9)	4.8 (2.9)	0.05	*N* = 8
I assess the patients health via telephone, email or other digital solutions	6.8 (2.5)	7.5 (1.6)	0.16	4.9 (2.7)	5.6 (2.8)	0.83	*N* = 13
I give health‐promoting advice and recommendations to patients via telephone, email or other digital solutions	5.5 (2.8)	6.0 (2.6)	0.57	5.0 (3.0)	4.8 (2.9)	0.79	*N* = 14
I give health promotion and illness preventive recommendations in accordance with national guidelines to patients	7.4 (1.9)	5.2 (2.6)	0.35	5.1 (2.7)	5.2 (2.8)	0.17	*N* = 4
I have a supportive ongoing dialogue with patients about their needs and wishes	8.6 (1.2)	6.6 (2.6)	0.25	4.2 (2.7)	6.1 (2.9)	0.47	*N* = 4
I focus on relatives’ need for support and guidance	8.2 (1.5)	8.0 (1.9)	0.02*	4.1 (2.7)	4.6 (2.9)	0.06	*N* = 5
I report all incidents in accordance with the actual patient safety system	8.4 (1.5)	7.6 (1.6)	0.16	4.4 (2.8)	5.7 (2.7)	0.10	*N* = 9

Note

Mann–Whitney U test. Level of significance 0.05. Significant differences marked *. The scale for self‐assessed competence ranges from 1 = bad to 10 = excellent. The scale for perceived need for more training ranges from 1 = no need to 10 = extensive need.

The items that showed significant differences were as follows: “I take patients’ mental health needs (mood swings, feelings of hopelessness, depression, etc.) into account when assessing and planning for the health and life situation of patients” (*p* = 0.04), “I take patients’ spiritual health needs (feelings of meaninglessness, existential needs, beliefs, fear of death, etc.) into account when assessing and planning for the health and life situation of patients” (*p* = 0.04), “I take patients’ physical health needs (illness, pain, disabilities, etc.) into account when assessing and planning for the health and life situation of patients” (*p* = 0.04), “I take patients’ social health needs (leisure activities, friends, financial situation, etc.) into account when assessing and planning for the health and life situation of patients” (*p* = <0.01) and “I focus on relatives’ need for support and guidance” (*p* = 0.02).

Nurses had the least self‐assessed competence on the item “I give health‐promoting advice and recommendations to patients via telephone, email, or other digital solutions.” Nurses perceived the most extensive need for further training on the item “I have knowledge of the interactions of various types of medication and what side effects they may cause for the patients I am responsible for” (Table 4).

Several of the items were reported as not covered in the nurses’ educational programme. The item most frequently reported was “I improve routines/systems that fail to meet the needs of patients at my workplace” (*N* = 19). Only one of the items was not reported uncovered by any of the nurses, namely “I adopt an ethical approach in my relationship with patients.”

Summing up the mean values of each of the items and dividing by item totals, the mean self‐assessed competence was 7.8 (*SD* = 1.1, median = 7.9) for nurses in primary health care, while tertiary care nurses reported a mean of 7.5 (S = 1.2, median = 7.7).

### 4.3 Job satisfaction

Comparative analyses, as assessed by the Mann–Whitney U test, showed no significant differences in nurses’ job satisfaction in primary and tertiary health care, respectively. An overview of nurses’ scorings on the JSS is presented in Table 5. The mean value for primary healthcare nurses’ job satisfaction was 5.9 (*SD* = 1.3, median = 6.0), and in tertiary healthcare nurses, it was 6.0 (*SD* = 1.1, median = 5.9).

**Table 5:** Results on the Job Satisfaction Scale

	Primary health care (*N* = 104)	Tertiary health care (*N* = 26)	*p*‐value
Responsibility	4.6/5.0 (1.3)	5.0/5.0 (1.4)	0.10
Variation in tasks	5.8/6.0 (0.9)	5.25/6.0 (1.6)	0.19
Colleagues	6.2/7.0 (1.3)	5.9/6.0 (1.1)	0.07
Physical environment	4.5/4.5 (1.5)	4.9/5.0 (1.5)	0.31
Possibility to use your abilities and skills	5.5/6.0 (1.3)	5.2/5.0 (1.5)	0.38
Total impression of your work situation	5.7/6.0 (1.1)	5.6/6.0 (1.3)	0.99
Feel free to make my own decisions	4.5/5.0 (1.3)	4.9/5.0 (1.5)	0.15
Acknowledgement from leaders and colleagues	4.9/6.0 (2.6)	4.5/4.0 (1.6)	0.18
Wages	2.8/2.0 (1.7)	3.3/3.0 (1.4)	0.08
Working hours	4.6/4.5 (1.7)	4.3/4.0 (1.5)	0.35
Considering how you feel right now, are you satisfied with your life, or are you mainly dissatisfied?	4.9/5.0 (1.4)	5.1/5.0 (1.4)	0.50

Note

Job Satisfaction Scale (JSS) scores: 1 = very dissatisfied. 7 = very satisfied. *N* = number of participating nurses.

### 4.4 Multiple regression

Multiple regression analyses showed that the type of ward, percentage of employment and years of experience as a nurse/from primary health care/from tertiary health care, continuing education or reported job satisfaction were not associated with any of the items or the mean score of the ProffNurseSAS self‐assessed competence or the perceived need for more training (Table 6). The internal consistency, as measured by the Cronbach's alpha, of both the ProffNurseSAS questionnaire and the JSS questionnaire in this study was 0.9 (=excellent).

**Table 6:** Multiple regression analysis of different factors’ association with self‐assessed competence and perceived need for further training

Factor	Self‐assessed competence		Need for further training	
	Standardized Beta	*p*‐value	Standardized Beta	*p*‐value
Type of ward	0.15	0.84	0.13	0.46
Percentage of employment	0.04	0.79	0.01	0.96
Years of experience as a nurse	0.17	0.64	0.02	0.95
Years of experience primary health care	−0.09	0.76	−0.17	0.58
Years of experience tertiary health care	−0.03	0.90	−0.32	0.18
Continuing education	−0.14	0.49	0.03	0.88
JSS	0.17	0.31	−0.12	0.47

Note

JSS: Job Satisfaction Scale score.

## 5 DISCUSSION

Findings show that nurses in primary health care had significantly more experience and a larger proportion had continuing education than nurses in tertiary health care. Five significant differences in self‐assessed competence between nurses working in primary versus tertiary health care were identified. Moreover, there were no differences in reported job satisfaction between primary and tertiary healthcare nurses. No associations between socio‐demographics or job satisfaction and self‐assessed competence/need for further training were found.

Our findings fill a gap in knowledge about nursing competence in the newly established municipal intermediate care or acute wards, in comparison with hospital wards. A 2010 review only identified two Norwegian studies concerning competence in community care (Finnbakk, Skovdahl, & Blix, 2012). A stepdown in competence outside institutions has been shown in studies comparing staff in home‐based care and staff in nursing homes (Hasson & Arnetz, 2008; Bing‐Jonsson, Hofoss, Kirkevold, Bjørk, & Foss, 2016; Finnbakk et al., 2015). Our findings may indicate that municipalities have used the years from the implementation of the Coordination Reform (CR) in well. Haukelien et al. (2015) explored nurses’ experiences with the implementation of the CR. Then, nurses claimed that the municipalities could not build sufficient competence and a professional infrastructure to meet the increasing complexity and number of patients in primary health care. In contrast, the significant differences in self‐assessed competence in our study indicated a higher self‐assessed competence in primary healthcare nurses: primary healthcare nurses had significantly higher self‐assessed competence on items such as considering patients’ mental, spiritual, social and physical health needs when assessing and planning for the health and life situations of patients, as well as focusing on relatives’ need for support and guidance. These items corroborate research on healthcare services with a patient‐centred approach (e.g., Bowie et al., 2015; Kitson, Marshall, Bassett, & Zeitz, 2012). Studies have also reported that patients experience patient‐centred care in the newly established municipal acute wards versus in hospitals (Leonardsen, Del Busso, Grøndahl, & Jelsness‐Jørgensen, 2016, 2017). This may indicate that nurses in primary health care have higher competence in providing patient‐centred healthcare services.

Nurses reported the lowest self‐assessed competence for the item “I give health‐promoting advice and recommendations to patients via telephone, email, or other digital solutions”; the highest perceived need for more training and/or education for the item “I have knowledge of the interactions of various types of medication and what side effects they may cause for the patients I am responsible for”; and the item “I give health promotion and illness preventive recommendations in accordance with national guidelines to patients.” This is important information for a society that searches for digital solutions to increase the efficiency of healthcare services. Studies have shown that the overall level of nurse competence as perceived by nurses is high (Bing‐Jonsson, Hofoss, et al., 2016; Istomina et al., 2011) and this is supported by our study. Nurses have assessed their competencies in managing situations and work roles as the highest and in teaching‐coaching and ensuring quality as the lowest (Istomina et al., 2011). Moreover, nurses have reported insufficient competence in areas like nursing measures, advanced procedures and nursing documentation (Bing‐Jonsson, Foss, Foss, & Bjørk, 2016), as well as in psychiatric and palliative nursing and certain technical skills (Furåker, 2012). Consequently, the findings in our study both support and add to earlier research. In addition, our findings reveal areas where nurses’ competence in both primary and tertiary health care could be improved. This knowledge is important when developing new healthcare services and in quality improvement initiatives.

Furthermore, areas that nurses reported as not covered by their nursing educational programmes were the “improvement of routines and systems,” participation in quality and competence development and creative learning. This lack of education was also related to evaluations of differential diagnoses, interactions and side effects of medication and giving health‐promoting advice to patients. Changes in the delivery of nursing have challenged nursing educators to seek innovative ways to ensure that their educational programmes produce competent practitioners. Schoneman, Simandl, Hansen, and Garrett (2013) found that curricula of participating colleges and universities did not address the necessary competencies in financial planning and management or leadership and systems thinking. Six areas of competence have been suggested for nursing education: patient‐centred care, teamwork and cooperation, evidence‐based practice, quality improvement, safety and informatics. Moreover, areas like ethical values, nursing skills, communication and inter‐personal skills have been emphasized (Bing‐Jonsson, Hofoss, et al., 2016; Kajander‐Ukuri, Salminen, Saarikoski, Suhonen, & Leino‐Kilpi, 2013; Kajander‐Ukuri et al., 2014). Hence, the findings in this study add to earlier research on areas that merit emphasis when planning and developing nursing educational programmes.

Both nurses in primary and tertiary health care had received training for the last two years. Since we do not have data from before the CR implementation for comparison, we cannot assume that this has changed due to the increasing challenges in healthcare services. Nevertheless, our findings show that nurses in primary health care also received training for specific patient cases, such as ventilators, tracheostomies, palliation or dialysis, which may indicate a greater emphasis on meeting the exacerbated severity and complexity of patients’ conditions in primary health care. This is supported by, for example, Henni et al. (2018), who emphasized a need for nurses in primary health care with advanced qualifications to adequately address the needs of frail older adults.

Regarding the scores on the JSS, work satisfaction has been inversely related to high levels of staff turnover (Karsh, Booske, & Sainfort, 2005; Sikorska‐Simmons, 2005; Van den Berg, Landeweerd, Tummers, & Van Merode, 2006). There were no significant differences in job satisfaction between nurses in primary (mean = 5.9) and tertiary health care (mean = 6.0). Job satisfaction was scored relatively high, and it was not associated with self‐assessed competence or the perceived need for more training and education. This is in line with a study that found that nurse competence influences job satisfaction and nursing performance (Ha & Choi, 2010) and may indicate that there will be competent nurses to provide primary healthcare services also in the future.

Moreover, findings show that demographic factors, such as years of experience, continuing education or job satisfaction, were not associated with self‐assessed competence or the perceived need for more training. This contradicts earlier studies, which identified nurse education, experience, professional development, independence, work satisfaction (Grönroos & Perälä, 2008; Istomina et al., 2011), professional group affiliation, workplace, age (Bing‐Jonsson et al., 2016), gender (Hamström et al., 2012) and marital status (Kim & Kim, 2015) as predictors of nurses’ self‐reported competence. Of course, a larger sample size may have detected similar associations.

Results on nurses’ self‐assessed competence, their perceived need for more training (or lack thereof) and their reported job satisfaction indicate high quality and safety in both primary and tertiary healthcare services. In this study, we could not confirm the suggested differences in quality, safety and competence in policy documents and media between the two levels of health care or the worries about enough competence to meet future needs.

### 5.1 Strengths and limitations

One limitation of this study is the lack of generalization of results due to the small sample size. A larger sample size may have given more significant differences. In some wards, the response rate was very high; in other wards, it was very low, and we cannot be sure that the sample is representative. In retrospect, we could have computed a power analysis and focused on including nurses accordingly, for example, by allowing the completion of questionnaires on professional development days. Moreover, we could have invited nurses from more hospital wards to better compare primary and tertiary health care. Nevertheless, the inclusion of so many wards representing both the central and rural parts of the county and both small and big units may strengthen the validity of our findings.

Nurses reported that the questionnaire was time‐consuming to complete. A shorter questionnaire could have increased the number of responders: a review study and meta‐analysis found that response rates were lower for longer questionnaires (Rolstad, Adler, & Ryd'en, 2011). Perhaps only nurses chose to participate who had high self‐assessed competence and little need for further training—or a positive attitude towards research and competence development. In addition, there is also a greater likelihood that those who felt the least competent refrained from taking part.

A limitation regarding the assumption that nurses in primary and tertiary health care have similar and sufficient competence is indicated in studies that indicate discrepancies between self‐assessments and observed performances (Baxter & Norman, 2011; Lauder et al., 2008). Further studies need to be conducted to evaluate the association between self‐assessed competence and actual knowledge and clinical skills.

The validity and reliability of this study is strengthened by the instruments used. The ProffNurseSAS, as well as the JSS, have been found to be valid and reliable (Andersen & Andersen, 2012; Finnbakk et al., 2015; Warr et al., 1979). The Cronbach's alpha was excellent in this study, which indicates proper internal consistency of the tools.

## 6 CONCLUSION AND IMPLICATIONS FOR CLINICAL PRACTICE

Findings show that nurses’ self‐assessed competence, perceived need for more training and job satisfaction were the same in primary and tertiary healthcare services. This indicates good quality, safe services for “the earlier hospital patients,” regardless of decentralization. The main areas nurses expressed a need for further training were, for example, in using digital solutions in communication with patients, health assessment of patients and knowledge about medication interactions. Hence, results in this study may be useful in quality improvement initiatives across healthcare levels, institutions and wards, as well as to educational institutions.

Further studies on nurses’ and other healthcare professionals’ competence in a larger sample would be useful to support this study's findings and to add even more knowledge to this under‐represented area of research. In addition, studies that support the link between healthcare personnel's competence and healthcare quality would be useful when planning educational and quality improvement initiatives.

## CONFLICT OF INTEREST

No conflicts of interest to declare.
